# Connecting Youth and Young Adults to Optimize Antiretroviral Therapy Adherence (YouTHrive): Protocol for a Randomized Controlled Trial

**DOI:** 10.2196/11502

**Published:** 2019-07-30

**Authors:** K J Horvath, R F MacLehose, A Martinka, J DeWitt, L Hightow-Weidman, P Sullivan, K R Amico

**Affiliations:** 1 School of Public Health University of Minnesota Minneapolis, MN United States; 2 Institute for Global Health and Infectious Diseases University of North Carolina at Chapel Hill Chapel Hill, NC United States; 3 Department of Epidemiology Rollins School of Public Health Emory University Atlanta, GA United States; 4 School of Public Health University of Michigan Ann Arbor, MI United States

**Keywords:** treatment adherence, antiretroviral therapy, adolescent, HIV, mobile apps, mobile phone

## Abstract

**Background:**

Despite intensive efforts to engage people living with HIV in the United States, less than half of the youth aged 13 to 24 years achieve viral suppression. There is a clear and continued need for innovative behavioral programs that support optimizing adherence among young persons with HIV.

**Objective:**

There are 3 phases of this project. Phase 1 involves conducting focus groups to obtain feedback from youth about an existing technology-based antiretroviral therapy (ART) adherence intervention. Phase 2 will be used to conduct beta testing with youth to refine and finalize the YouTHrive (YT) intervention. Phase 3 is a randomized controlled trial (RCT) to test the efficacy of the YT intervention among youth living with HIV (YLWH).

**Methods:**

In phase 1, we will conduct 6 focus groups with approximately 8 youths (aged 15-19 years) and young adults (aged 20-24 years), each in 3 US cities to obtain (1) feedback from YLWH about the *look and feel* and content of an existing adult-focused Web-based ART adherence intervention and (2) suggestions for adapting the intervention for YLWH similar to themselves. Phase 2 will involve updating the existing intervention to include features and functionality recommended by YLWH in phase 1; it will conclude with beta testing with 12 participants to gain feedback on the overall design and ensure proper functionality and ease of navigation. For phase 3, we will enroll 300 YLWH in 6 US cities (Atlanta, Chicago, Houston, New York City, Philadelphia, and Tampa) into a 2-arm prospective RCT. Participants will be randomized 1:1 to YT intervention or control group. The randomization sequence will be stratified by city and use random permuted blocks of sizes 2 and 4. Participants randomized to the control condition will view a weekly email newsletter on topics related to HIV, with the exception of ART adherence, for 5 months. Participants randomized to the YT intervention condition will be given access to the YT site for 5 months. Study assessments will occur at enrollment and 5, 8, and 11 months post enrollment. The primary outcome that will be assessed is sustained viral load (VL), defined as the proportion of participants in each study arm who have suppressed VL at both the 5- and 11-month assessment; the secondary outcome that will be assessed is suppressed VL at both the 5- and 11-month assessment between drug-using and nondrug-using participants assigned to the YT intervention arm.

**Results:**

Participant recruitment began in May 2017 for phase 1 of the study. The data collection for aim 3 is anticipated to end in April 2020.

**Conclusions:**

The efficacy trial of the YT intervention will help to fill gaps in understanding the efficacy of mobile interventions to improve ART adherence among at-risk populations.

**Trial Registration:**

ClinicalTrials.gov NCT03149757; https://clinicaltrials.gov/ct2/show/NCT03149757 (Archived by WebCite at http://www.webcitation.org/73pw57Cf1)

**International Registered Report Identifier (IRRID):**

DERR1-10.2196/11502

## Introduction

### Background and Study Aims

Youth aged between 13 and 24 years accounted for 22% of all new HIV infections in the United States in 2015. The majority of these infections (81%) occurred among gay and bisexual young men [1]. Less than half (44%) of youth living with HIV (YLWH) in the United States are virally suppressed, which is a well-recognized critical factor in individual health and noninfectiousness. Drivers of viral suppression for all people living with HIV (PLWH) include access to effective antiretroviral therapy (ART), initiation of and persistence with ART regimens, and consistent ART adherence. Predictors of ART adherence among youth are multifactorial and include medical (eg, side effects and dissatisfaction with the medical team), logistical (eg, forgetting and inconvenience), and psychological (eg, depression, lack of support, and perceived stigma) barriers [2]. In addition, substance use among adolescents and young adults remains high and is associated with ART nonadherence [3,4], making it an important, although underutilized [5], target for ART interventions with YLWH. For these reasons, there is an ongoing need for innovative programs that leverage current communication channels to foster social support for ART adherence behaviors.

Social support has been conceptualized as a basic human need, acts as a buffer to stress, is a fundamental coping strategy, and serves to engender understanding and assistance [6]. Greater social support is associated with improved behavioral and health outcomes for adults with HIV, including serostatus disclosure [7] and lower sexual risk-taking among gay male couples [8]. Among children and adolescents, having a *buddy system* for remembering to take ART is associated with greater adherence [9]. For these reasons, a leading group of medical and behavioral science experts has recommended that peer support may be considered to improve ART adherence outcomes [10].

Traditional, in-person peer-support ART promotion interventions have been conducted for adolescents [11] and adults [12,13]. For example, Simoni et al randomized 224 adult HIV-positive patients at a public HIV specialty clinic in Seattle, Washington, to receive either in-person peer-support, pager messaging, both in-person and peer messaging, or usual care [13] for a 3-month period. Those receiving the peer intervention had higher self-reported adherence at the immediate postintervention assessment, although intervention effects diminished at later assessment periods (with the final assessment point at 9 months) [13].

Technology-based ART adherence approaches have proliferated in recent years [14-16] because of the widespread adoption of technology across sociodemographic groups [17], their ability to reach a broad audience, and their low implementation costs [18]. Youth are especially appropriate candidates to receive technology-based ART adherence interventions. Youth were early and heavy users of technology [19]. Recent data from the Pew Research Center showed that most (95%) US teens are online, 80% own a desktop or laptop computer, and 78% and 37% own a mobile phone or smartphone, respectively [20,21]. Most (92%) 18- to 29-year-olds own a smartphone [22], allowing mobile access to the internet for most youth. Technology provides ways to create virtual support networks that bypass geographic boundaries, thereby providing access to supportive others that may otherwise be unavailable because of geographic or stigma barriers. A recent analysis of message posts from a closed Facebook group for patients who were part of a young adult (aged 16-25 years) HIV program showed that members provided high levels of emotional and network support and moderate levels of informational support [23]. However, most computerized ART adherence interventions [24,25] are individually delivered, and most fail to leverage peer-to-peer interactivity that has come to symbolize Web 2.0. Given the increasing use of social media as important and influential communication channels and the high demands of social identity development during adolescence and early adulthood, interventions that leverage support networks for HIV-positive youth may be an important avenue to address HIV care outcomes.

The *Thrive With Me* (TWM) intervention was developed by members of this study team and leverages online peer support to improve ART adherence among adult men who have sex with men (MSM) residing in New York City, New York [26]. TWM is a responsive website that adjusts to the size of the device (computer, pad or tablet, or phone) on which it is being viewed. Responsive websites, sometimes referred to as *Web apps*, can have a similar appearance and functionality as a native app but are less costly to develop and can be viewed across multiple devices. TWM is a peer-support, tailored information, and self-monitoring ART adherence intervention grounded in the Information, Motivation, and Behavioral Skills (IMB) model [27,28]. A pilot of TWM was conducted between February and April, 2010, to assess its feasibility, acceptability, and preliminary efficacy among adult MSM primarily recruited online in the United States [29]. MSM (n=123; mean age 43 years; 64% white, non-Hispanic; and 16% used drugs, excluding marijuana, in the past 30 days) were randomly assigned to receive either the TWM intervention (n=66) or no intervention (n=57) for 2 months. Assessments occurred at baseline and 2- and 3-month follow-up periods. Moreover, 90% of participants were retained at the 3-month follow-up assessment, and those randomized to TWM reported high levels of perceived information and system quality, usefulness, and overall satisfaction of the intervention. Adherence scores were not significantly different for the full sample. However, there was some evidence of greater improvement in timely dosing (ie, taking ART within 2 hours of the usual dosing time [*P*<.10] and taking ART correctly with food [*P*<.05]) among the intervention group than the control group. Improvement in ART adherence outcomes was most pronounced for current (ie, ≤30 days) drug-using MSM among whom the TWM intervention arm reported significantly higher overall ART adherence (*P*=.02) and ART taken correctly with food (*P*=.01) than those in the control condition. Currently, TWM is being assessed in a large (n=400) efficacy trial of MSM residing in NYC [26].

This study describes the protocol for a randomized controlled trial (RCT) of the YouTHrive (pronounced “Youth Thrive” or abbreviated as YT) Web-based intervention, which is being adapted from the TWM intervention. The primary goal of YT was to improve ART adherence and HIV treatment outcomes among viremic YLWH. YT uses a multicomponent package of peer-to-peer social support, tailored HIV and ART information, and self-monitoring to achieve this goal.

The aims of the research include the following:

The primary objective is to assess the efficacy of YouTHrive (YT) in a 2-arm RCT (n=300) to sustain suppressed viral load (VL) among YLWH, compared to an HIV information-only control condition.

H1: A higher proportion of participants in the YT intervention arm than in the information-only control arm will have undetectable VL at both the 5- and 11-month follow-up time points.

The secondary objective is to assess whether YT is more beneficial for substance-using than nonsubstance-using YLWH.

H2: Among YLWH in the YT intervention arm, a higher proportion of substance-using YLWH will demonstrate VL suppression at both the 5- and 11-month follow-up time points compared to nonsubstance-using YLWH.

### Theoretical Basis for YouTHrive

**Figure 1:**
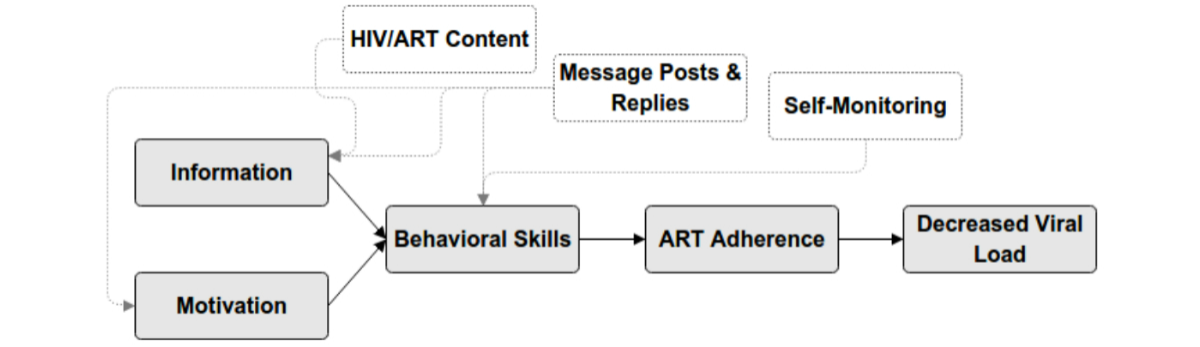
Thrive With Me intervention components and the Information, Motivation, and Behavioral skills model. ART: antiretroviral therapy.

The IMB model proposes that health behavior and behavior change results from being well and accurately informed, having the personal and social motivation to engage in the behavior, and having the appropriate behavioral skills and self-efficacy to use them [27,30]. The associations between core YT intervention components (described in detail below) and the IMB model components are shown in Figure 1. The IMB model has been used to predict risky sexual behavior among adolescents in Los Angeles, CA [31] and has been used as the theoretical basis of adolescent risk reduction interventions [32]. The model has also been evaluated and supported in studies of ART adherence using clinic-based samples of adults in Puerto Rico [28], Italy [33], and Mississippi [34] and among a community-recruited sample of HIV-positive MSM in the United States [35].

## Methods

### Ethics Statement

The institutional review board (IRB) at the University of North Carolina Raleigh Durham, NC, is the IRB of record for all participating institutions and subject recruitment venues (SRVs) participating in the study. It will review all procedures outlined in this protocol. Procedures for phase 1 of the study (outlined below) have been approved (UNC IRB 16-3136). A waiver of parental consent was obtained for participants who are aged 15 to 17 years. The study is registered as a clinical trial (Clinical Trials # NCT03149757).

### Design

**Figure 2:**
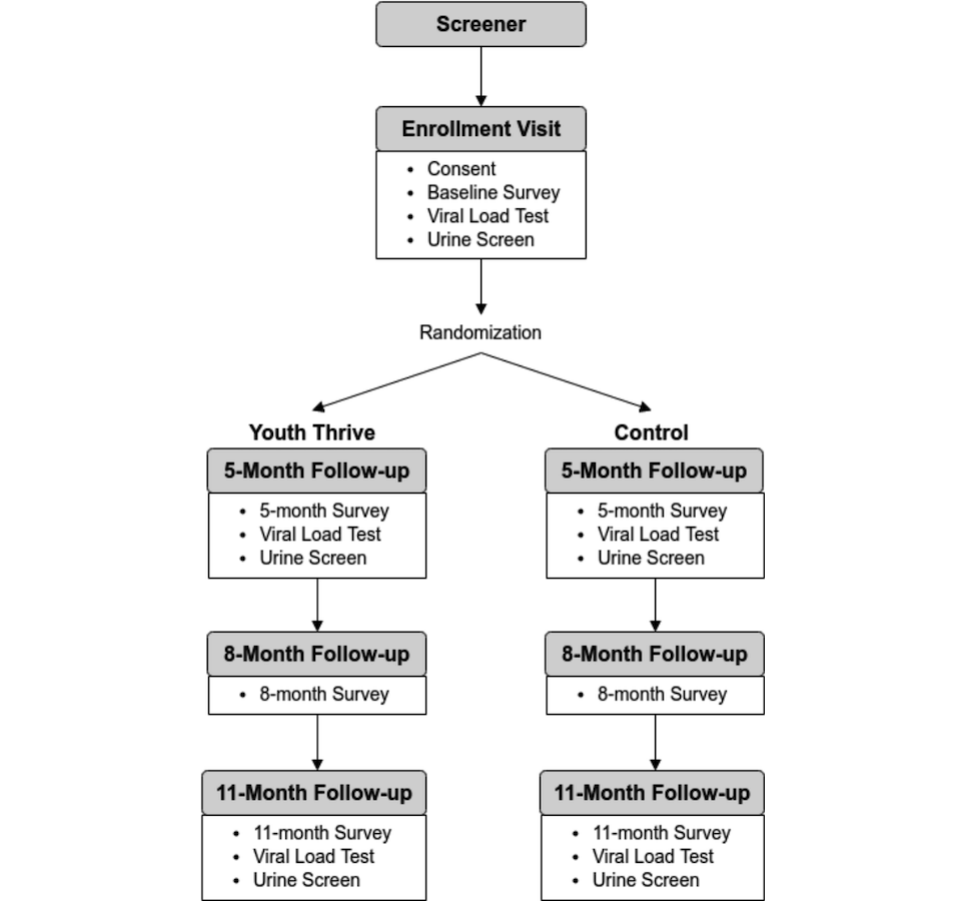
YouThrive intervention flow.

We will evaluate the YT intervention in a randomized controlled efficacy trial (see Figure 2). There are 3 phases of the YT study:

#### Phase 1: YouTHrive Intervention Adaptation

We will conduct 6 focus groups with approximately 8 youth (aged 15-19 years) and young adults (aged 20-24 years), each at 3 SRVs (Chicago, New York, and Houston) to obtain (1) feedback from YLWH about the *look and feel* and content of the original TWM intervention and (2) suggestions for adapting the intervention for YLWH similar to themselves. Focus groups will be transcribed verbatim, and a content analysis will be assisted by the analytic core (AC). Feedback from the focus groups will be used to inform phase 2.

#### Phase 2: YouTHrive Adaptation and Beta Testing

We will work with our technology partner, Radiant Creative Group (RCG), to adapt the TWM intervention to include features and functionality that are identified in the first phase of our research. Beta testing with 12 participants will be conducted to get feedback on intervention design (ie, the overall *look and feel* of YT), functionality (ie, whether YT is functioning properly), and navigability (ie, users can easily navigate features) to finalize all features and components of the intervention for phase 3.

#### Phase 3: Randomized Controlled Trial to Test Efficacy of YouTHrive

YLWH (n=300) will be recruited by staff from 6 SRVs located in Atlanta, Chicago, Houston, New York (the Bronx borough), Philadelphia, Tampa, and (see Recruitment Strategy below). Persons who are interested in the study will be screened for eligibility using an online screening survey. YLWH who meet all inclusion criteria (detailed below) will complete an enrollment visit, which may be on the same day as screening or on a different day.

At the enrollment visit, participants will complete an in-office baseline computer-assisted survey instrument (CASI) and will be randomized at survey outset to either intervention or control. YLWH will then complete in-person overview and training on condition-specific site use. Youth assigned to the YT intervention will be shown example webpages of the intervention, will be given basic training on how to navigate the intervention, and will be given the opportunity to ask questions they have about the website. Control condition assigned participants will be shown example control arm webpages. Intervention and control conditions are detailed below.

The active intervention and control period will last 5 months. We will employ up to 5 YLHW to be active in the YT site at first until there are 10 participants randomized to the YT intervention arm. This is to ensure that youth entering the intervention arm early have others with whom they can interact and not requiring participants to wait until other participants are enrolled. During the 5-month intervention period, YT intervention arm participants will have continuous access to the YT site, whereas youth in the control condition will receive an email newsletter with HIV-related information once per week.

Follow-up assessments will be conducted at 5-month (ie, immediate postintervention; follow-up 1 in the clinical setting), 8-month (follow-up 2 in an online-only CASI), and 11-month (follow-up 3 in the clinical setting) time points. Follow-up visits 1 and 3 will include an in-office administered CASI, a blood draw to test for detectable VL, and a urine screen for drug use, whereas follow-up 2 is an online CASI only.

### Exit Interviews

At the 5-month visit, up to 20 participants randomized to the YT intervention arm will participate in a remote video-based interview conducted by study staff. The purpose of this interview is to elicit feedback on their experiences using the YT site, any technical difficulties encountered, and how the site could be further improved. Participants will be selected for interviews using purposive sampling based on level of engagement with the site (ie, high engagement vs low engagement relative to other users). All interviews will be audio recorded for transcription and analysis. Study staff will track activity levels and select participants for the interview.

### Participants and Sample Size

We plan to enroll 360 HIV-positive adolescents and young adults in this study. Up to 48 participants will be recruited for focus group discussions (with the goal of 8 per group) for phase 1, 12 participants will be recruited to conduct beta testing of the YT intervention in phase 2, and 300 participants (n=150 YT and n=150 control) will be recruited to participate in the YT RCT in phase 3. Participants who are pregnant at the time of screening or who become pregnant during the study period will not be excluded from the study.

Inclusion criteria for each phase of the study are described below:

#### Focus Groups Inclusion Criteria

The focus group inclusion criteria are as follows: (1) self-reporting 15 to 24 years of age at screening; (2) HIV positive; (3) currently taking ART medication; (4) self-reporting missed medication doses in the past month or detectable VL or no VL in past 12 months; (5) engaged in care at the Chicago, Houston, or New York City SRV; (6) owns a cell phone; and (7) proficient in English as determined by study staff (as the intervention will be built in English). Focus groups will be stratified by age (1 focus group of youth aged 15 to 19 years; 1 focus group of age 20 to 24 years per site) to ensure that the perspectives of both youth and young adults are explored.

#### Beta Testing Inclusion Criteria

Beta testing inclusion criteria are as follows: (1) 15 to 24 years of age at the enrollment visit; (2) HIV-positive status (medical chart verified); (3) HIV clinical care in Atlanta, Chicago, Houston, New York City, Philadelphia, or Tampa area; (4) currently prescribed ART (medical chart verified); (5) medical chart–verified detectable VL (above the lower limit of detection for the clinical assay) within 52 weeks of enrollment date and an ART prescription for at least 90 days before this VL test date; (6) English-speaking; (7) internet and short message service (SMS) messaging access for the beta testing period (approximately 2 weeks); (8) available to meet with site project staff in person for the first research appointment; and (9) available to meet with University of Minnesota project staff for a remote (ie, telephone or videoconference) feedback interview.

#### Randomized Controlled Trial Inclusion Criteria

RCT inclusion criteria are as follows: (1) aged 15 to 24 years at the enrollment visit; (2) HIV-positive status; (3) residing in Chicago, Houston, NYC, Philadelphia, Atlanta, or Tampa area and respond that they will be available to meet with SRV staff for visits at baseline, and 5-month and 11-month follow-up assessments; (4) evidence of a current ART prescription; (5) English-speaking; (6) anticipated continuous internet access and SMS messaging for the intervention period (approximately 5 months); and (7) has or is willing to create an e-mail address to use during the study period; (8) not a member of an iTech Youth Advisory Board (YAB); and (9) meets one of the following medical-chart verified or self-reported criteria: (a) one or more detectable VL test result (above the lower limit of detection for the clinical assay if medical-chart verified) in the past 12-months while on ART for at least 3 months, (b) having failed to show up for or missed 1 or more scheduled HIV care appointment in the past 12 months, (c) last HIV care visit was more than 6 months ago, or (d) self-reporting less than 90% ART adherence in the past 4 weeks. Persons enrolled in another ART adherence intervention research study at the time of screening will be excluded from participation.

### Study Recruitment

Participants for all phases of this study may be approached and recruited in 1 of 2 ways: (1) in the SRV HIV clinic or (2) in the community. Recruitment procedures may vary slightly depending on the SRV and study phase.

#### Subject Recruitment Venue HIV Clinic-Based Recruitment

Youth who are patients at the SRV will have his or her medical chart reviewed to assess for potential eligibility (eg, age, HIV status, on ART, and detectable VL in the past 12 months) and, if an appropriate candidate, will be approached for recruitment. Potential participants will be informed of the nature of the study, the information to be collected, and the evaluations and assessments that are involved. Those who express interest in the study will be required to be screened using a computer-based screener on a desktop computer or tablet (located at the SRV) to determine if they meet all inclusion criteria (including self-reported criteria that may indicate a high potential for problematic adherence). If eligibility criteria are met, the youth will be invited to enroll in the study immediately or at a later date if they are not available then.

#### Community Recruitment

Community outreach may bring in youth who are patients at other local HIV clinics or who are out of HIV care and would benefit from the SRV clinical care program. New patients can be considered for this project if they meet eligibility criteria. Outreach to community-recruited youth can include contacting potential participants via telephone or electronically (e.g., text message or email), attendance at community venues where youth not in HIV care spend time or through targeted ads on widely used social media channels (eg, Facebook and Grindr). Youth who are approached for recruitment in person or contacted remotely (e.g., telephone or email) will be asked to complete the online screener and, if preliminarily eligible, scheduled for an enrollment visit. The receipt of clinic services will not depend on expressing interest or enrolling in the YT study (ie, clinical care will be based on usual clinic requirements).

### Randomization for Phase 3

Once participants are enrolled and complete the baseline assessment, they will be randomized 1:1 to YT intervention or control group, based on a randomization sequence developed by the AC statistician and programmed into SurveyGizmo. Study staff will not be blinded to which arm youth are randomized; however, because both conditions are active (ie, youth receive content with which they may interact), we have confidence that youth will not be aware of whether they are assigned to the intervention or control condition. The randomization sequence will be stratified by city [36] and use random permuted blocks of size 2 and 4. Although the proportion of YLWH who report recent substance use will not be used to direct recruitment efforts or target enrollment numbers, we do anticipate high rates of alcohol, marijuana, and other illicit drug use based on analyses of substance use in prior Adolescent Medicine Trials Network for HIV/AIDS Interventions (ATN) studies (87% for ATN 125; 76% for ATN 086-106) [3,37]. The percentage reporting substance use (defined as any alcohol, marijuana, or other illicit drug use) at baseline will be examined once half of the total target enrollment is reached (n=150). If substance use is reported by less than 50% of participants, we will re-examine recruitment efforts and the inclusion criteria to determine if changes are needed to bolster recruitment of substance-using YLWH.

### Youth Advisory Board

We will consistently work with Youth Advisory Boards (YABs) at each SRV to elicit feedback from YLWH regarding our language, design, and gaming choices that would be most interesting and relevant to youth. YABs, at most SRVs, meet monthly to review study materials and provide feedback to study teams during phase 2 and as needed in phase 3.

### Intervention

In the RCT, intervention participants will have access to the full YT website for 5 months. The YT intervention will be developed as a safe space for sharing information and helping youth feel empowered and supported to make healthy choices around living with HIV. To be available to answer questions and enforce community standards (eg, no hostile exchanges), the YT website is moderated by trained research staff. Moderating includes reading through posted comments on the wall each day and identifying posts that are concerning (eg, suicidal ideation, pleas for assistance, and potentially hostile comments to other users). The study protocol and procedures manual for moderating details the exact steps taken for each potential situation. Note that posts are not delayed or held until *cleared* for posting. Rather, the moderator reviews posted material and acts accordingly. This is to retain the immediacy of posting, which users of social media largely expect. Similar to the TWM intervention for adult MSM [35], there are 3 core components in the YT intervention, which are described below.

#### Message Posting and Receiving

The YT homepage will consist of an interface for participants to asynchronously interact with one another through message posting. Unlike widely used social networking platforms such as Facebook, participants will view all posts on 1 shared feed (vs individual feeds or direct messaging). Other users may comment on a post as well as use reaction buttons (eg, thumbs up and Superman symbol). Message posting is the primary social support component of the intervention, as it allows participants to directly and voluntarily interact with one another in a similar manner as a face-to-face peer support group (see Multimedia Appendix 1).

#### Antiretroviral Therapy and HIV-Related Content

Adherence and HIV content (see Multimedia Appendix 2) will be presented as “Thrive Tips” on the YT site. Youth in the YT intervention arm will receive approximately 3 Thrive Tips each day. Thrive Tips can include (1) brief tips about how to live with HIV and better manage medication adherence, (2) videos or links to videos of youth discussing challenges to ART adherence and ways to overcome them, and (3) image-based content such as memes or infographics. Study staff created approximately 300 Thrive Tips total, with two-thirds dedicated to theory-based adherence barriers, and the remaining one-third considered *Grab Bag* tips that include content about general well-being while living with HIV (stress management, dealing with HIV while in school, dating and relationships, and healthy sexuality). All participants in the intervention arm will receive every Thrive Tip in the first half of the 5-month intervention period. Tips that reflect a participant’s unique adherence information, motivation, and adherence self-efficacy barriers, as assessed from the baseline survey responses, will be identified with an icon (eg, a fire symbol) to encourage greater engagement with tips tailored to youth’s specific adherence barriers. At the halfway point, youth in the YT condition will retake the IMB-related adherence information and motivation scales and the Adherence Self-Efficacy Scale (ASES) to update their adherence barriers profile, and all of the Thrive Tips will be shown a second time for the last half of the intervention period.

Content for the Thrive Tips is curated and created by the study team with input from the YABs. Aim 1 focus groups will be used to guide revisions of Thrive Tips from the TWM intervention to include youth-oriented language, images, and videos as well as content that is inclusive to all genders and sexual identities (because the TWM intervention is designed specifically for adult MSM). As participants are in the intervention for longer periods, they accumulate more saved Thrive Tips. They can search and revisit all accumulated Thrive Tips through a keyword search.

#### Medication Adherence and Mood Self-Monitoring

At setup, participants will be guided in setting up their profile with their current ART medication and next HIV care appointment, both of which can be updated throughout the intervention period. Youth will have the ability to self-monitor whether they took their dose(s) of ART each day as well as indicate their daily mood by selecting the representative emoji, through the *My Check-in* feature on the YT interface. Youth will have the ability to retrospectively input their adherence and mood for up to 72 hours. Underneath, a calendar will be displayed that reflects their personal adherence behaviors through color coding (eg, blue-shaded days are days that medications are taken, and red-shaded days represent days for missed doses) with overlaid emojis that indicate their mood for the day to promote greater insight of the connection between participants’ adherence behaviors and mood states (see Multimedia Appendix 3).

#### Goal Setting and Monitoring

Youth will view an interface called “My Journey” that leads them through steps to set and self-monitor 1 or more goals. Goals will include, but will not be limited to, those about living with HIV and ART adherence. For example, a youth may identify that she or he would like to improve school performance. Participants could choose the goal of determining which time is best to take their HIV medications if the medications interfere with their thinking or could choose the goal of asking for help with their homework from a classmate, teacher, tutor, or parent/guardian. In addition, youth will be given the option of writing in their own goal. Once a goal is set, youth will indicate when they would like to achieve that goal (eg, next week and next month). The goal will then appear as an active goal in which youth will self-monitor how much progress they have made toward that goal with a 7-point scale from “thinking about starting” to “journey complete.” Youth will be asked if they would like to share on the community wall that they have completed a journey. Participants may start and complete as many journeys as they wish during the study period.

#### Weekly Short Message Service Engagement Message

All participants will receive a weekly SMS text message that prompts and encourages them to visit the YT site. SMS text messages will be designed to engage youth with the different aspects of the site, including Thrive Tips (*“Extra, extra! Read all about it! Log in here to see today’s* tip.”), goal setting and monitoring (“You’re halfway through your time on YT. Take a minute to update your Journey”), adherence and mood monitoring (eg, “Have you had time to check in today? Log into the YT site now.”), advancing point levels (eg, “Step up your game! Log into the YT site and see how to earn more points.”), and trending topics on the community wall (eg, “People on the YT site are talking about [insert trending topic]. Come join the conversation!”).

#### Game Mechanics

The YT intervention uses points that accumulate as youth use intervention components to reinforce engagement with the site. As points accumulate, youth move through higher levels (ie, “levelling up”) during the intervention period, which unlocks new features of the site (eg, new avatar choices and color theme choices) when a new level is achieved. Points are earned through posting to the YT feed (wall), responding to other users’ comments, setting new goals, clicking on a Thrive Tip, and other actions that may be taken in YT. Youth will be able to view the number of points and their current level as part of their profile.

#### Control Condition

The control condition consists of 21 brief informational text and graphic-based webpages that will be released weekly (1 webpage per week for approximately 5 months), similar to a *newsletter*. This weekly newsletter will be provided to participants as an email with a link they can click on to open the newsletter on their mobile internet browser. The newsletters will contain information on topics related to living with HIV (eg, disclosure of HIV at school and work) and devoted to improving general well-being (eg, managing depression) but not specifically about ART adherence. Informational content will be chosen from a list of topics reported to be of most interest and relevance by participants in phase 1 focus groups and YLWH serving on YAB.

### Outcomes

Study outcome measures and the timing of their administration are provided in Table 1. Outcomes are described below.

**Table 1:** Study outcome measures and administration schedule.

Measure	Baseline	5-month assessment	8-month assessment	11-month assessment
HIV RNA viral load	X^a^	X	—^b^	X
Sociodemographics	X	X	X	X
Technology use	X	X	X	X
System Usability Scale	—	X	—	—
Electronic health literacy	X	—	—	X
Intervention acceptability	—	X	—	—
Antiretroviral therapy (ART) regimen and engagement in HIV care	X	—	—	X
Self-report ART adherence	X	X	X	X
Adherence information and motivation	X	X	X	X
Adherence self-efficacy and adherence support	X	X	X	X
Substance use	X	X	X	X
Sexual behavior	X	X	X	X
Mental health	X	X	X	X
HIV stigma	X	X	—	X
Social support	X	X	X	X
Social support in YT^c^	—	X	—	—
Emotional regulation	X	—	X	—

^a^Measure included at the assessment.

^b^Measure not included at the assessment.

^c^YT: YouTHrive.

#### Primary Outcome

Sustained undetectable VL is the primary outcome, defined as having an undetectable VL at both the 5- and 11-month time points. VL was chosen as the primary outcome as it is one of the most objective and reliable indicators of ART adherence [38]. VL data will be collected either through chart review, if a VL test was conducted and reported in the medical chart within a 30-day window from the study visit date, or through blood draw taken as part of the research protocol at the time of the assessment. Undetectable VL will be measured at the standard level of detectability at each SRV (eg, if the SRV typically uses <40 copies/mm as undetectable, then participants are reported undetectable at that SRV if they are below that level). In addition, the date of the VL test, the VL assay type, the lower and upper limits of the test, and the source of the VL test (eg, laboratory report and clinician’s notes) will be noted.

#### Demographic Factors

Common demographic factors will be collected, including date of birth, race or ethnicity, zip code, sex assigned at birth, sexual identity, outness of sexual identity to others (family, friends, and medical providers), education, employment status, health insurance, family income, housing stability, and history in the criminal justice system.

#### Self-Reported Antiretroviral Therapy Adherence Variables

We will use the 3-item Adherence Scale developed by Wilson et al [39], which asks how well job participants did at taking their HIV medicines the way they are supposed to, how often in the past 30 days they took their HIV medicines in the way they were supposed to, and a Visual Analog Scale in which participants can report the percentage of HIV medicines they took in the past 30 days. Youth will also be asked to report how many days they missed at least one dose of their HIV medicines in the past 30 days.

To assess theoretically derived ART adherence strengths and barriers, the information and motivations scales from the IMB ART Adherence Questionnaire (IMB-AAQ) [40] will be completed by participants. The IMB-AAQ assesses adherence-related information (9 items) and personal and social motivation (10 items) on a 5-point Likert scale. In addition, participants will complete the HIV ASES, which is designed to measure self-efficacy for adherence to HIV treatment plans, included but not limited to HIV medications [41]. Respondents are asked 12 questions to assess their confidence to carry out important treatment-related behaviors to adherence to treatment plans. Responses range from 1 (cannot do it at all) to 10 (certain can do it). Higher scores indicate higher adherence self-efficacy.

Finally, we will ask youth to indicate the kinds of adherence support they received from their clinic, provider, friends, or family during the intervention period. Adherence support may include electronic dose monitoring devices, dose reminder texts, alarms, phone calls, pillboxes, reminders, and help from family and friends and support groups.

#### Antiretroviral Therapy Regimen and Engagement in HIV Care

The following items will be abstracted from the medical chart of participants: (1) date of HIV diagnosis, (2) current and recent HIV medications, (3) past 12 month HIV care visits, (4) missed HIV care appointments in the past 12 months, and (5) future HIV care appointments from the date of abstraction.

#### Substance Use

Substance use will be assessed in several ways. First, youth will complete a urine screen at baseline, immediate postintervention, and 6-month postintervention time points to assess for the following illicit substances: cocaine, methamphetamines, marijuana, opiates, and phencyclidine, using a generic 5-panel screening test (model WDOA-554; DrugTestsInBulk website, West Hills, CA). The estimated detection periods for the test used are 2 to 4 days for cocaine and opiates, 5 to 30 days for marijuana, 1 to 3 days for ecstasy, and 3 to 5 days for methamphetamine. Second, participants will complete an adapted version of the National Institute on Drug Abuse (NIDA)-Modified Alcohol, Smoking, and Substance Involvement Screening Test (ASSIST). The ASSIST is a 9-item questionnaire developed by the World Health Organization and addiction researchers to screen for all levels of problem or risky substance abuse [42]. The ASSIST will be used to assess the frequency of use and associated problems for tobacco, alcohol, cannabis, cocaine, amphetamines (including methamphetamine and ecstasy), inhalants, sedatives, hallucinogens, and opioids.

#### Mental Health

Depression and anxiety symptoms will be assessed using the 8-item Patient Health Questionnaire (PHQ-8 [43]) and the 7-item Generalized Anxiety Disorder (GAD-7 [44]) scales. Youth will first be asked the first 2 items for each scale; those who report having some depressive or anxious symptoms (≥3 across the 2 PHQ items and/or ≥3 on the 2 GAD items) will be asked to complete the reminder items for the scales.

Emotional regulation will be assessed using the Emotional Regulation Questionnaire, which is designed to assess individual differences in the habitual use of 2 emotional regulation strategies: cognitive reappraisal and expressive suppression [45]. Participants will indicate their tendency toward reappraisal (6 items) and suppression (4 items) through a 7-point Likert scale, ranging from 1 (strongly disagree) to 7 (strongly agree). Higher scores indicate greater use of the emotional regulation strategy.

HIV stigma will be assessed using the stigma scale developed by Earnshaw et al [46]. Designed to measure HIV stigma mechanisms defined by the HIV Stigma Framework, the measure includes 3 subscales: internalized HIV stigma, anticipated HIV stigma, and enacted HIV stigma [46]. Items are rated on a 5-point Likert-type scale with higher scores indicating greater stigma.

#### Relationship Status and Sexual Behavior

To assess relationship status, youth will be asked to define their primary relationship status (eg, I am casually dating, I have a boyfriend or girlfriend, and I am single) and for those reporting being in partnership, whether they have sex outside the primary relationship as well. Sexual behavior is assessed by asking whether they have ever and in the past 3 months engaged in vaginal, anal, or oral sex. If they reported having sex in the past 3 months, youth will be asked how frequently (from none of the time to all of the time) they use a condom during insertive and receptive anal sex and vaginal sex.

#### Social Support

The Patient-Reported Outcomes Measurement System (PROMIS) short-form versions of the Social Relationships Scales will be used to measure perceived social isolation and social support [47]. PROMIS, a National Institutes of Health initiative, uses rigorous processes to develop and test item banks that measure physical, mental, and social health components [48]. The 5 Social Relationships Short-Form Scales, each with 4 items, measure social isolation and social support domains including companionship, emotional support, informational support, and instrumental support [47]. For youth randomized to the YT condition, emotional support, informational support, and social isolation within the YT intervention will be assessed. As instrumental support cannot be provided within this virtual intervention, we will not ask about this type of support within YT.

#### Technology Adoption and Use

Technology use questions and items assessing participants’ attitudes toward technology were taken from items developed by the Pew Research Center’s Internet, Science, and Tech initiative. Youth in YT will be asked to report device ownership; how they access the internet; which smartphone operating system they use and how they pay for service; how many hours a day they spend on the internet; how often they use mobile apps; frequency of internet use for social, sex-seeking, work, and health-seeking activities; the frequency with which they use social networking service (eg, Facebook and Instagram); and whether and how they may have faced discrimination while looking for partners on online venues. In addition, the 8-item eHealth Literacy Scale will be used to assess participants’ perceptions of their skills for using information technology (ie, the internet) for health [49].

#### Intervention Ease of Use, Acceptability, and Satisfaction

Youth in both study arms will be asked to rate the ease of use of their respective activities (either the YT intervention or the active control condition) using the System Usability Scale (SUS) [50]. The SUS is a 10-item measure that asks participants to rate on a 1 (strongly disagree) to 5 (strongly agree) scale how much they agree with statements about the ease with which they were able to navigate the intervention (eg, “I found YouTHrive unnecessarily complex” and “I found the various functions in YouTHrive to be very well integrated”). Participants will also answer questions to assess information quality (eg, “The information on YouTHrive is accurate”), perceived usefulness of the information (eg, “YouTHrive helps me quickly find information and support for healthy living”), and overall satisfaction with the intervention (eg, “Overall, I am very satisfied with YouTHrive”). We also ask youth to rate their respective intervention (YT or control) on information quality (eg, “I trust the information on [project name]”), usefulness (eg, “[name of project] helps me to quickly find information and support for healthy living”), and overall satisfaction (eg, “Overall, I am satisfied with [name of project]”) using items adapted from Horvath et al [35]. Finally, we will collect qualitative data on youths’ experiences by asking participants to state what they like most and least about the intervention, what was most memorable about it, and what features would make it better.

#### User Engagement

Intervention use data will be collected during the active trial period to assess user engagement with the intervention. Standard use data include (1) participant identification (ID) number, (2) study arm, (3) log-in date and time, (4) type of device used, and (5) total duration of the session. Intervention use data will include the following variables reflecting peer-to-peer interaction: (1) date of post, (2) original post content, (3) participant ID of original post, (4) content of replies to the original post, and (5) participant ID of each reply. Additional user engagement variables collected are (1) frequency of wall posts by participant ID, (2) number of comments by participant ID, (3) number of Thrive Tips viewed, (4) number of tailored Thrive Tips viewed, (5) number of Thrive Tips marked as *favorites*, (6) number of SMS engagement messages clicked on to take the user to the site, (7) number of days ART adherence reported, (8) number of mood responses reported, (9) number of goals set, (10) number of times progress toward goals is reported, (11) total number of active intervention days, (12) number of times the participant updated their outward-facing profile features, and (13) total points earned.

### Analysis

#### Phase 1

Audio recordings from each focus group will be transcribed and checked for accuracy by a member of the AC. Next, all transcriptions will be reviewed by at least three members of the study team. Participants’ overall feedback and their suggestions for changes to the overall look and feel, content, and specific features will be compiled in a document and reviewed by investigators. Recommendations for changes to the user interface will be prioritized and presented to RCG to guide the development of the beta version of YT.

#### Phase 2

Interviews with YLWH during beta testing will be audio recorded and reviewed by study team members. Feedback from youth will be recorded in a spreadsheet by intervention component, with suggestions for improving the overall design and content recorded separately. A beta testing report will be compiled by Dr Horvath and reviewed by the investigator team and RCG. The report will include a list of common navigation problems by intervention component, suggestions for improvement in design and content by intervention component, and recommended design and content improvements for the overall site. The study team and RCG will review the beta testing report to prioritize modifications that need to be made, given the importance of the change to the user experience as well as what changes are possible, given time and budget constraints. They will then agree on final modifications to YT.

#### Phase 3

Stata version 15 (StataCorp) and SAS version 9.4 (SAS Institute) were used for power calculations and will be used for all analyses. The primary study outcome is HIV viral suppression at both 5 and 11 months of follow-up, measured as undetectable VL based on the standard level of detectability. The primary statistical test of intervention efficacy for YT will be the comparison between intervention and control arms of the proportion of participants with undetectable VLs at both the 5-month and 11-month follow-up time points using a differences in proportions test and confidence intervals. If there is evidence of baseline imbalance between intervention arms for important predictors of viral suppression, we will fit logistic regression models that adjust for those covariates.

As a secondary aim, we will investigate whether there is greater benefit from the YT intervention for substance-using participants compared with nonsubstance-using participants. We will use the same modeling approach described above to address this aim. First, we will examine the association between the intervention and viral detection separately among those who did and did not self-report current (since the last visit) substance use (ie, yes/no for problematic alcohol use and/or illicit drug use). Second, to formally test whether there is an interaction between intervention arm and substance use, the models described above will be refit including an interaction term between substance use and interaction arm. Interactions will be evaluated on the additive scale. We will carefully examine the distribution of potential confounders of the substance use and VL association and adjust for them as necessary. As mentioned previously, we will adjust for covariates where appropriate.

The models described use logistic regression to model the outcomes. We will use estimates from these models to report prevalence differences and ratios. However, alternative (log-linear and Poisson) models may be explored to allow easy interpretation of parameters in the presence of common outcomes. In the event of loss-to-follow-up among study participants, we will perform sensitivity analyses of an alternative outcome. We will define an additional outcome where a failure is defined as either detectable VL or loss-to-follow-up. The analyses described above will be repeated with this alternative outcome. All of the models mentioned above can be modified to accommodate missing values in the outcome or covariates over time without dropping participants. Although attempts will be made to limit missing data, in the event that this occurs, we will carefully examine patterns of missingness. Multiple imputation will be implemented, as needed, to deal with missing covariate data.

Finally, we will examine models that include covariates that quantify the degree of site usage and which components were used. The additional outcome of self-reported ART adherence will be examined. The outcome will be defined as the percentage of ART taken in the past 30 days. Differences in this proportion by study arm will be evaluated using the same approach as described above. To explore the effects of the YT intervention on the intermediate theory-based processes of change, we will use the IMB-AAQ informational and motivational scales, adherence self-efficacy, and social support measures at each time point. These scales will be included as outcome variables in linear regression models to test the main effect of intervention arm.

### Incentives

The method for compensation will be determined separately by each site and approved by each SRV’s IRB. Participants at all sites will be compensated with the following cash or cash equivalent. Focus group participants will be compensated US $30 and refreshments during visits. Beta test participants will be paid US $25 at the first visit and US $25 at the second visit. RCT participants will be compensated US $50 at the enrollment visit and the 5-month and 11-month follow-up visits. Participants will be compensated US $20 for completing the 8-month remote online survey. Participants who complete the exit interview at the 5-month follow-up visit will be compensated an additional US $50. If a participant is unable to go into the clinic to complete a follow-up study visit, the Web-based CASI survey could be completed on his or her own. SRV study staff will be notified when a participant has completed a CASI survey on his or her own, and the compensation will be provided to the subject. Compensation can be mailed to participants, if allowed at the site.

### Power and Sample Size

This study is powered to detect a meaningful effect for the main aim that there will be a difference between the proportion of virally suppressed participants in the intervention and control arm. The sample will consist of people who are and are not virally suppressed at baseline. We assume that 30% of participants will not be virally suppressed at baseline. Among this 30% who are not virally suppressed, we assume 30% of them will be virally suppressed at all follow-up times in the control arm and 47% will be virally suppressed at all follow-up times in the intervention arm (for a difference of .17). Among the 70% who are virally suppressed at follow-up, we assume 80% will be virally suppressed at all follow-up times in the control arm and 95% will be virally suppressed in the intervention arm (for a difference of 0.15). The average risk of the viral suppression in the control arm is therefore 65% and the average risk in the intervention arm is 80.6% (a risk difference of .16 or a risk ratio of 1.24). Assuming a type 1 error rate of 5% and a 1:1 allocation of participants to the treatment and control arm, we have 80% power to detect this difference of .16 or risk ratio of 1.2 if we enroll 256 participants (128 per arm). We assume there will be 15% loss to follow-up and will attempt to enroll 300 participants to account for this.

## Results

Participant recruitment began in May 2017 for phase 1 of the study. The data collection for phase 2 is expected to be completed in June 2018 and for phase 3 in October 2020. Final results are anticipated for April 2021.

## Discussion

There are a number of challenges to the YT clinical trial. First, it will require a multipronged effort to meet our recruitment goals, especially for younger YLWH. To address this, the research team will work with recruitment venues to reach out to as many of their current clinic population who meet eligibility requirements as possible. In addition, we will implement community and social media recruitment strategies to identify potential participants who may either be out of HIV care or are not current patients at recruitment clinics. Second, we do not provide smartphones or other Web-enabled devices to study participants but rather require that participants own or have access to a Web-enabled device. This may restrict participation by lower socioeconomic status youth and young adults on the one hand but will increase the potential for scale-up on the other hand. Moreover, providing devices to study participants is cost prohibitive and, therefore, not feasible within this protocol. That said, given that nearly all (95%) of teens have access to a smartphone [51], we believe that we will be able to capture most youth and young adults who are eligible for this study. Third, given high rates of mental health problems among HIV-positive youth [52-54], medical and psychological services must be available during the study period. We will implement SRV-specific protocols to assess and provide referrals to medical and psychological services in the event that a participant should report a need for these services or experience any adverse reactions resulting from study procedures.

With these limitations in mind, YLWH face numerous intrapersonal, social, structural, and cultural challenges, many of which impact their engagement in HIV care and, ultimately, interfere with their ability to adhere to ART. Given that the HIV epidemic in the United States has shifted toward younger ages of infection [55] and the high proportion of HIV-positive youth who are not virally suppressed [2], innovations in programs to promote and sustain adherence behaviors over time are critically needed.

There is a growing use of technology as a medium to reach youth with HIV with adherence interventions [56-60]; however, a number of critical questions remain. First, although technology use is highly prevalent among youth [20], there is still a lack of understanding for best practices to engage them in technology-based interventions. This is particularly true with respect to racial and ethnic minority persons [61]. Formative research that asks PLWH to identify which features of technology-based intervention approaches they believe would be most engaging can be useful [62]; however, intervention studies that assess the association between use of different intervention components with primary outcomes are needed to identify those components that are most engaging and effective [63]. Second, although the literature provides results from efficacy trials of computerized [24,25] and text message [64] interventions, trials of mobile ART adherence interventions remain poorly represented in the evidence base [65]. There is a need to rigorously test mobile interventions (including native app and Web app interventions) to advance research in this area. Third, it remains unclear how best to incorporate technology-based ART adherence interventions into clinical care and into the lives of youth over long periods. Further research is needed to understand if these and similar types of interventions are best delivered continuously or whether they should be available on demand, as youth experience periods of hardship that impact their adherence behaviors. Similarly, it is not clear whether technology-based interventions are most effective when integrated with clinic electronic health care records or whether concerns about privacy, availability, and autonomy create demand for these types of programs that lie outside of the health care system. The YT study described here will begin to provide answers to some of these important questions.

## Appendix

Multimedia Appendix 1 Screenshots of intervention components.

Multimedia Appendix 2 YouTHrive beta testing guide.

Multimedia Appendix 3 YouTHrive focus group discussion guide.

## Abbreviations

AC: analytic core
ART: antiretroviral therapy
ASES: Adherence Self-Efficacy Scale
ASSIST: Alcohol, Smoking, and Substance Involvement Screening Test
CASI: computer-assisted survey instrument
GAD: Generalized Anxiety Disorder
IMB: Information, Motivation, and Behavioral Skills
IMB-AAQ: Information, Motivation, and Behavioral Skills ART Adherence Questionnaire
IRB: institutional review board
MSM: men who have sex with men
PHQ: Patient Health Questionnaire
PLWH: people living with HIV
PROMIS: Patient-Reported Outcomes Measurement System
RCG: Radiant Creative Group
RCT: randomized controlled trial
SMS: short message service
SRV: subject recruitment venue
SUS: System Usability Scale
TWM: Thrive With Me
VL: viral load
YAB: Youth Advisory Board
YLWH: youth living with HIV
YT: YouTHrive
