# Supplementary material for: Connecting Youth and Young Adults to Optimize Antiretroviral Therapy Adherence (YouTHrive): Protocol for a Randomized Controlled Trial
Source: JMIR Res Protoc. 2019 Jul 30;8(7):e11502. doi: 10.2196/11502 (PMC6691670; doi:10.2196/11502)

APPENDIX 1. Screenshots of Intervention Components

Message Posting and Receiving

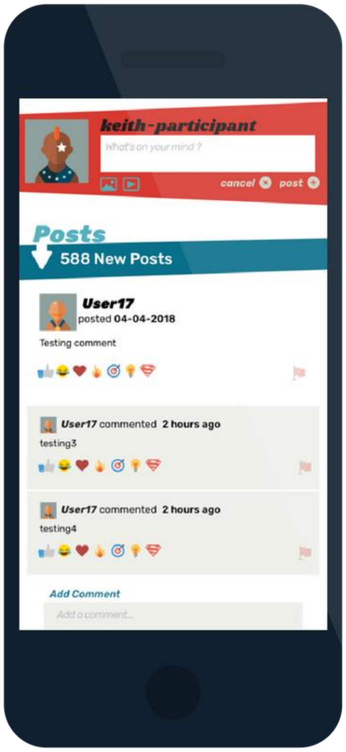

Adherence and HIV Content

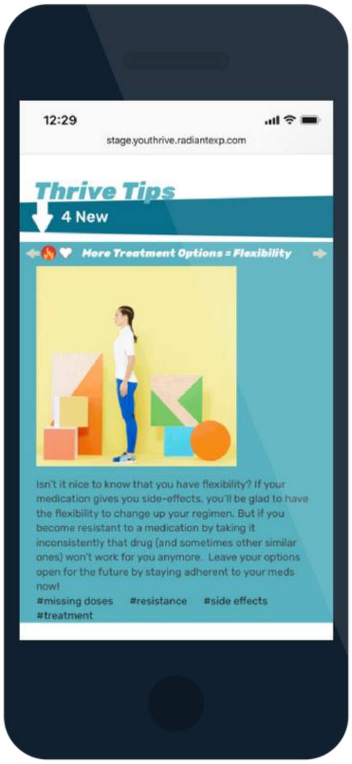

Medication Adherence and Mood Self-Monitoring

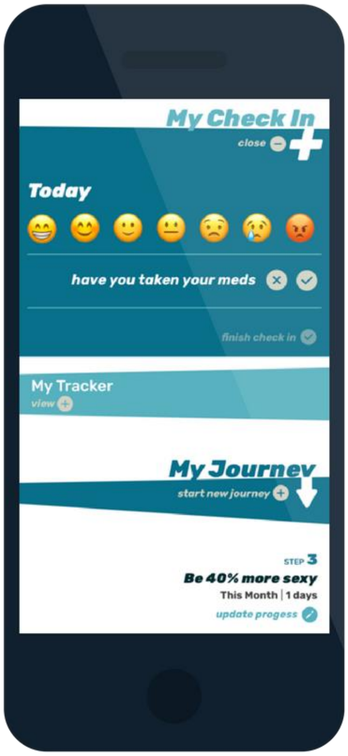

Goal Setting and Monitoring

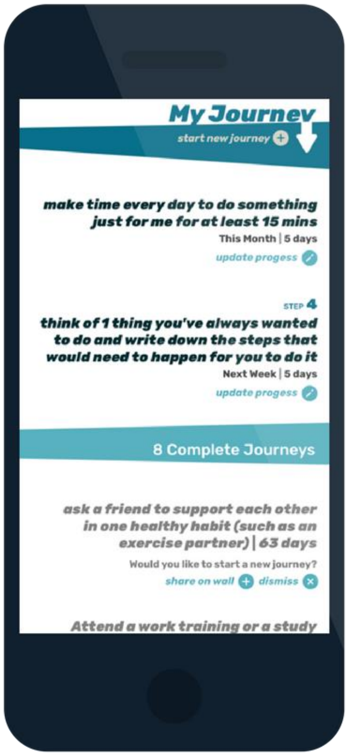

User Profile

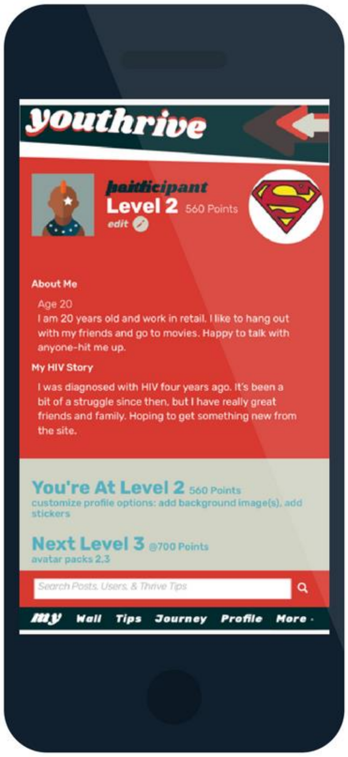

Supplement: Multimedia Appendix 1 [file resprot_v8i7e11502_app1.pdf]
