# Supplementary material for: Connecting Youth and Young Adults to Optimize Antiretroviral Therapy Adherence (YouTHrive): Protocol for a Randomized Controlled Trial
Source: JMIR Res Protoc. 2019 Jul 30;8(7):e11502. doi: 10.2196/11502 (PMC6691670; doi:10.2196/11502)
Supplement: Multimedia Appendix 2 [file resprot_v8i7e11502_app2.pdf]

YouTHrive (YT): Aim 2 - Usability Testing Semi-Structured Debriefing Guide and Sample Questions

Introduction:

<u>Speak date and time for voice recorder.</u>
Thank you for evaluating the YT website. I have a few additional questions I would like to ask to better understand your experience with using the site over the past couple of weeks. Feel free to look at the notes you took or to look at the site on the assessment room computer when giving an answer.

Sample Questions and Prompts:

First, I would like to know about how you accessed the site.

1. What types of devices did you use to access the YT site (phone and phone type, computer and computer type, tablet and tablet type)?
2. (If multiple types of devices) Did you use one type more than another? Please describe if you had a better experience using one type of device over another.

Next, I'd like you to think about logging into the site. You most likely logged in for the first time in-person at the clinic. Did you have any issues logging in after that first session?

1. Thinking back, did you have further impressions of the log-in process that you would like to share?

During your first meeting, you were also asked to set up an aspirational journey to follow.

1. What did you like about setting up your path? If you want, feel free to look at the journey section of the site to refresh your memory.
2. What did you dislike about setting up your path?
3. What could have made it easier to set up your path?
4. How did you feel about the tone of this section of the site?

Let's discuss the text messages a little bit.

1. Did you receive a text message each week?
2. What did you think about the content of the text messages?
3. What did you like about getting the weekly text messages?
4. What did you dislike about getting the weekly text messages?
5. Is there anything you think we could do differently to improve the weekly text messages?

Great, thanks. Now I want to walk through different parts of the site and get your feedback on them.

1. Staying on the “My Profile” tab, what did you think about the different options for profile fields?
2. What did you like about choosing your avatar?
3. What did you dislike about choosing your avatar?
4. Did you enter anything in the About Me section? What did you like about the About Me section?
5. What did you dislike about the About Me section?
6. Did you like the other profile fields you could fill out? Why or why not?
7. What could be done to make the profile page more personal or interesting?

Let’s look at the points you have earned through using the site.

1. What are your thoughts on the points system?
2. Do you particularly like any of the features unlocked by earning points?
3. Do you particularly dislike any of the features unlocked by earning points?
4. Did you feel motivated by the points or unlockable features?
5. How do you think other people will be motivated by points or unlockable features?
6. What could be done to make the points system more personal or interesting?

Let’s navigate to the About pages.

1. What are your thoughts on our types of About pages?
2. What kind of information did you like in the About pages?
3. Did you find anything unclear in the About pages?
4. What could we do to improve the About pages?

Great, thank you. Let’s now navigate to the Getting Started tab.

1. What are your general thoughts on having tips on Getting Started?
2. What could we do to make this clearer?
3. What could we do to make this more interesting?

Okay, now we’re going to return to the Wall/Home page starting with the daily Thrive Tips.

1. Did new Thrive Tips appear every day on your wall?
2. Did you have any difficulties finding the Thrive Tips on your wall?
3. What did you like about having Thrive Tips delivered to your wall?
4. What did you dislike about having Thrive Tips on the wall?
5. Did you receive any tips that were highlighted for you? How did you recognize them?

6. Can you please describe how you would find a particular Thrive Tip from a previous day?

Thinking about the content in the Thrive Tips….

1. Do any Thrive Tips stick out in your mind as having content that you really liked?
2. Do any Thrive Tips stick out in your mind as having content that you really disliked?
3. Did you have any issues loading videos or images? Please describe.

Turning back to the Wall/Home page…

1. Can you please describe if you had any issues posting status updates to the site? How about posting pictures of videos?
2. What did you think of what other users were posting?
3. What would have made you post more?
4. Did you notice the achievements/badges on the wall? Can you please describe your thoughts on the achievements that you saw on the wall?

The final bit on the site that I want you to navigate to is the part where you can track your adherence and mood.

1. Were you able to track your adherence and mood at least once?
2. What did you think about any reminders you received to track your adherence and mood?
3. Was it easy or difficult to figure out where to track your adherence?
4. What did you think of the ability to track trends over time in mood and adherence?
5. Is there anything we could do to make the tracking easier to use?
6. How do you think the adherence and mood tracking could be helpful to other users?

To finish, I want to ask some general questions about your experience with the site.

1. What was most memorable about the YT site?
2. What was most frustrating about the YT site?
3. Would you use this site if it were available to you? Why or why not?
4. Would you recommend this site to your friends? Why or why not?
5. What else would you like to tell us about the site that we haven't talked about?
6. Is there any other advice that you may have for us?

Thank you very much for your thoughtful insights and time! We very much appreciate your help.
