# Supplementary material for: Connecting Youth and Young Adults to Optimize Antiretroviral Therapy Adherence (YouTHrive): Protocol for a Randomized Controlled Trial
Source: JMIR Res Protoc. 2019 Jul 30;8(7):e11502. doi: 10.2196/11502 (PMC6691670; doi:10.2196/11502)
Supplement: Multimedia Appendix 3 [file resprot_v8i7e11502_app3.pdf]

Youth Thrive Discussion Guide

Introduction

Hi everyone. Welcome to the focus group. Thank you for taking the time to be here.

[Introduce team members]

Before we start, we want to have a few group rules:

- We want to make this a safe space for everyone. Please only use the name you provided when you filled out the form earlier. When you talk, please state the name you want to use today so we know later on who was speaking. I'll remind you about that as we begin.
- People have different opinions about things. Please share your opinion and respect the opinions of others.
- What is said in this room stays here. Please respect the right of everyone to privacy.
- We only have a short time together, so if we're spending too much time on a topic, I'll ask to move to the next topic. I'm not intending to be disrespectful, but I want to make sure we're getting to all of the topics.
- Remember to have fun. This is meant to be informal and we're just asking for your opinion. We'd love to hear from you, but will not force anyone to talk.
- It's ok to respond to each other's comments. If someone says something that you have some thoughts on, please say something.
- Finally, we want to make sure that it's ok to audio record this focus group. This is only because we can't take notes fast enough to capture everything you're sharing with us. So this will allow us to go back and review what we talked about. When you talk, please only use your first name or any name you'd like us to use for you. Please don't use your last name. Is it ok to begin the recording?

Any questions?

Ice Breaker

- To give you some information about why we've asked you to be here today…we've created a website that adults living with HIV are using right now to help them live with HIV and to help them take their HIV meds.
- We want to now figure out how we should change the website to be more engaging and helpful to youth with HIV. So we're asking you for your input.
- Say something about why this is important to me.
- Icebreaker
    - Name for today
    - Age
    - Something you want to do this summer

Overview of Focus Group

Here's what I'd like to cover today:

1. First I'll ask a couple questions about how you use technology in your daily life. This will help us know what's important to you with respect to technology.
2. Then most of the time we will spend looking at the website I talked about before and getting your feedback and thoughts on it.

Technology Use and Medication Adherence

Let's begin…

3. Everyone uses technology in different ways.
    - What sites do you go to most often?
    - What social networks do you use most often?
        - What do you like best about the social networks you use?
    - What devices do you use most (phone, tablet, computers)?
        - Are these private – that is only you have access to them - or shared, like with a family member or friend?
    - Is there anything else that you think we should know about how you use technology in your daily life?

Feedback on the TWM Website

That's really helpful. We're going to change topics now.

- Now we want to get your feedback on a website that we've developed.
- This website is called "Thrive With Me"
- TWM is currently being tested with adult men and we want to get feedback from you about how we can change it to be useful for **young people of all genders and all identities**
- A few things that we think are important to understand this site:
    - When users sign up for this study, they come into a clinic and take a survey. Some of the information from the survey will link to user's personal account. I'll show you later how that works.
    - All users log in the first time with help from a research assistant at the clinic. Users get trained on how to the use the site.
- What I want to do today is to walk through the different sections of the site and get your feedback on each part about what you like, don't like, and what you would suggest we add to be more useful or engaging to you.
- Any questions about what we're going to do?

- [log onto https://demo.thrivewithme.net/ User: giorgio; PS: thriveguy]

Log-in Page

- Here is the login page
    - Clearly identifies this as a research study
    - You have to enter a username and password

[log into the demonstration site]

Home Tab – this is the first page that people see when they log in

- Important things you should know:
    - This site can be viewed on a laptop like this or on a smartphone.
    - Shared Feed
    - It's anonymous
    - People can talk about what they want but the site is moderated to make sure it's a safe space for everyone.
- **What's on your mind**
    - This lets you to post messages to the group like Facebook, but everyone can see all posts. So no one is writing directly on your wall and there is no private messaging.
        - Text
        - Links
        - Gifs/images
        - You Tube or Vimeo videos
        - [post: Hey everyone. I'm new to this. I'm working on taking my meds on time. Any advice would help!]
        - [scroll through the feed to show them what different posts look like]
- **Comments box**
    - After you post something, you can see if others have commented on your post here [show comments bar]
    - But users don't get a text or email notification that someone commented on their post – they just need to check the site and see who commented on it.
- **Badges, Points, and Leaderboard**
    - How many points they've earned by interacting with the site.
        - The more points, the higher level the user goes up
        - Badges – users receive badges for doing things on the site. To see what badges are given for an action, just click on it.
        - On the leaderboard, users see who is leading for the week on the left and which users have earned the most points overall.
- Daily Thrive Tips - We'll get back to this feature after we talk about the other things on this page.

Questions:

Let’s talk about the “Community posting” feature

4. What is your overall impression of the ability of users to post to the group and comment on other users posts?
   - What questions do you have about it?
5. What kinds of thing do you see yourself posting to the group?
   - Topics related to HIV
   - Topics not related to HIV
6. Do you think you’d post different things than you would to Instagram or Facebook?
7. **Go to comment box**: What do you think about the ability to see who has commented on your post or liked your post in this box?

Now let’s talk about earning points, badges and the leaderboard:

8. What is your overall impression of this feature?
   - What questions do you have?
9. Looking at the right side of the screen, how would points, badges or the leaderboard motivate you to use the site?
   - How do you view having this on a site? Do you see it as something fun just for you or a competition or something else?
   - Are there other game-like features (i.e. art features like Snapchat vs. competition) that you think should be included?

Daily Thrive Tips

- These are brief articles, videos or memes/gifs, or links about living with HIV and medications. These will change every day.
    - On home page:
        - You will get about 4 new Thrive Tips every day.
        - You can save your favorites by clicking the heart [click one of them]
    - The Thrive Tips that accumulate each day will show up and be searchable under the tab “Thrive Tips”
        - [Go to Thrive Tips tab – show search options]
            - Search all of the thrive tips by broad topics
            - Search those that are recommended just for you, shown by having a blue triangle in the corner
            - Search by only the ones you favorite
            - Search by tags

Questions:

10. What is your overall impression of the Thrive tips?
    - What questions do you have?
11. What would make you want to open a Thrive Tip?
    - Do you prefer a certain type of thrive tip? In other words, would you prefer to get your thrive tips as videos, images, memes, or text?
    - What is the longest video you would likely watch?

**Topic**
Self care (tips for managing your physical, mental, and emotional health, such as ways to relax and take time for yourself)
Dealing with HIV when you’re a student in school
Breaking down stereotypes about who is HIV+
Portrayals of people living with HIV in the media (TV, movies, etc.)
Depression and anxiety
Treatment fatigue or when you are tired of having to deal with chronic illness
What it is like to be a young person with a chronic illness
Self love
Dating and disclosure when living with HIV
Making sex safer when living with HIV

Sexual pleasure and wellbeing
Isolation and loneliness
Exercise and physical activity
Sleep

- What other topics, besides tips for taking your meds, would you like to see included in these Thrive Tips?

Text Messaging Reminders

- Each day users receive a text message reminding them to take their medications. [Show participants what a typical text message looks like]
- Once users get the reminder, they are asked to say whether they took their meds or didn't over text.
- Finally they are asked how they're feeling with 6 different options.
- All of that information goes into our system and then users can see for each week how they did in taking their mediations and how they were feeling that day.
- Users can also say on what days they used or did not use drugs or alcohol.

Questions

12. What is your overall impression of the medication reminder text messages?
    - What questions do you have?
13. How do you think a daily text message may help you take your meds?
    - What do you think about the actual wording of the messages?
    - Do you feel like the mood choices reflect are how you would talk about how you feel?
    - What are your thoughts about receiving daily text messages?
        - Would you prefer to receive text messages less frequently?
        - Would you prefer to receive a daily text message to check in about whether you took your meds that day and your feelings, or would you prefer to answer those things right on the site and not receive any text messages?

14. What is your overall impression being able to see how you did in taking medications on the days of that week and your mood?
    - What questions do you have?
    - Are there other ways you can think of about how we could display this?
15. How would it be helpful to look back at prior weeks to see how you were taking your meds, and how you were feeling?
    - Would you like to be able to download this information to your phone or some other device? How would you use this if you were able to download it?
    - How would you share this weekly check-in with your doctor, nurse, or case manager?
    - Are there other features of the weekly check-in you think we should add or remove? For example, do you like being asked about alcohol and drugs, or are there other things that would be more important to keep track of?

My Profile

- Profile picture (we don't allow users to use their own picture, but give them a few dozen options of avatars)
- Write something about yourself
- Talk with me about
- Your badges and points
- All of your posts that you've been involved with
- You can edit your profile by clicking here [click Edit Profile]
    - Edit your password
    - Profile picture
    - Medication Reminders
        - Change the time you want to receive it
        - And whether you want the standard message, that includes the word "dose" or the alternative message that does not include language about medications, dose or anything else meds related.
        - [Show examples of text messages now]

Questions

16. What is your overall impression of this feature?
    - What questions do you have?
17. What do you think about how the avatars look?
    - How could we improve the avatars to make them feel more unique or like they represent you?
18. What suggestions to you have for making the "Talk to me about…" section more useful, interesting, or fun to you?
19. What ideas to you have about things we should add to the profile page to make it more interesting or fun?

Finally, we won't discuss it much, but we also have tabs that:

- Tell users about the study and the people on it [show **About** tab]
- A glossary of terms that users might find on the site that they want to know more about [show **Glossary** tab]
- A tab that shows users how to use the site [show **Getting Started** tab]

Questions:

20. **Any feedback on these pages that you'd like to share?**

Feedback About TWM

21. What are your overall thoughts about the TWM site?
    - What is most memorable about it?
    - What is the confusing parts about it?
    - How do you think you would use it?
    - What would you change about it?
22. Thank you so much for taking the time to share your thoughts today. Is there any final advice on ways to make this site better?

Thanks!

[turn off recording]

[collect notes from FG members]
